# Microbiological and cytological characterization of coelomic fluid from three captive endangered amphibian *Gastrotheca* species with edema syndrome: preliminary analysis

**DOI:** 10.1186/s13104-019-4846-3

**Published:** 2019-12-16

**Authors:** Anahí Oleas-Paz, Ana Cecilia Santamaría-Naranjo, Maira Rojas-Carrillo, Andrés Merino-Viteri, Alexander Genoy-Puerto

**Affiliations:** 1 Escuela de Medicina Veterinaria, Facultad de Ciencias de la Salud, Universidad de Las Américas, Vía Nayón S/N, 170503 Quito, Pichincha Ecuador; 2 Laboratorios Multidisciplinarios de Ciencias Biológicas y Químicas, Universidad de Las Américas, De Los Colimes y Avenida de los Granados, 170125 Quito, Pichincha Ecuador; 3 Museo de Zoología (QCAZ), Escuela de Ciencias Biológicas, Pontificia Universidad Católica del Ecuador, Av. 12 de Octubre 1076 y Roca, 170523 Quito, Pichincha Ecuador

**Keywords:** Marsupial frogs, Edema fluid, *Klebsiella ozaenae*, *Burkholderia pseudomallei*, *Enterococcus* sp.

## Abstract

**Objective:**

Edema syndrome is highly prevalent but under researched in captive frogs around the world. The objective of the present study was to characterize at a basic microbiological and cytological level of the bacteria of the edema fluid of 20 individuals of the genus *Gastrotheca* to determine the presence of possible anaerobic and aerobic bacteria.

**Results:**

Fourteen types of bacteria were identified in the edema fluid, 12 of them at the species level (*Pasteurella haemolytica*, *Hafnia alvei*, *Enterobacter agglomerans*, *Aeromonas hydrophila*, *Pseudomonas fluorescens*, *Burkholderia pseudomallei*, *Salmonella arizonae*, *Enterobacter gergoviae*, *Enterobacter sakazakii*, *Yersinia enterocolitica*, *Klebsiella oxytoca,* and *Klebsiella ozaenae*) and two at the genus level (*Enterococcus* spp. and *Streptococcus* spp.). The most frequently identified cells were lymphocytes (37.7% in females and 46.4% in males), erythrocytes (23.5% in females and 17.5% in males) and neutrophils (4.2% in females and 2.8% in males). Finally, no relationship was found between the data obtained and the sex of the individuals studied.

## Introduction

Amphibians are distributed worldwide in different ecosystems. However, there is currently a marked decline in the amphibian population [1–3]. Ecuador is one of the countries that faces this decline [4], and the *Gastrotheca* genus is one of the threatened amphibian genera [5–7].

Because of this decline, in Ecuador, conservation projects have been developed to mitigate the decline. The “Balsa de Sapos” Conservation Initiative is one of those projects. A large number of amphibian species in captivity has led to the realization of scientific studies concerning pathologies and diagnostics of diseases, such as edema syndrome [8, 9].

Edema syndrome is a disease that affects innumerable amphibian species and has a high prevalence among frogs in captivity [10, 11]. Causes of edema syndrome vary from bacterial septicemia and fungal or viral infection to metabolic disorders and dietary and husbandry deficiencies [9, 10, 12–17]. Clinical microbiology approaches, as applied in this study, improve the knowledge and management of this health problem in captive amphibian populations.

The following study presents initial microbiological and cytological characterizations of the edema fluid of individuals of the genus *Gastrotheca* from the “Balsa de Sapos” Conservation Initiative of the Pontificia Universidad Católica del Ecuador. The species studied were *Gastrotheca* (*G*.) *litonedis*, *Gastrotheca riobambae* and *Gastrotheca pseustes*, all of which are endemic to Ecuador and assigned to different categories of extinction risk [5, 6].

This study helps to improve the health management of the animals in this institution and to provide the information needed to create health management plans in captive breeding institutions.

## Main text

### Methods

**Fig. 1:**
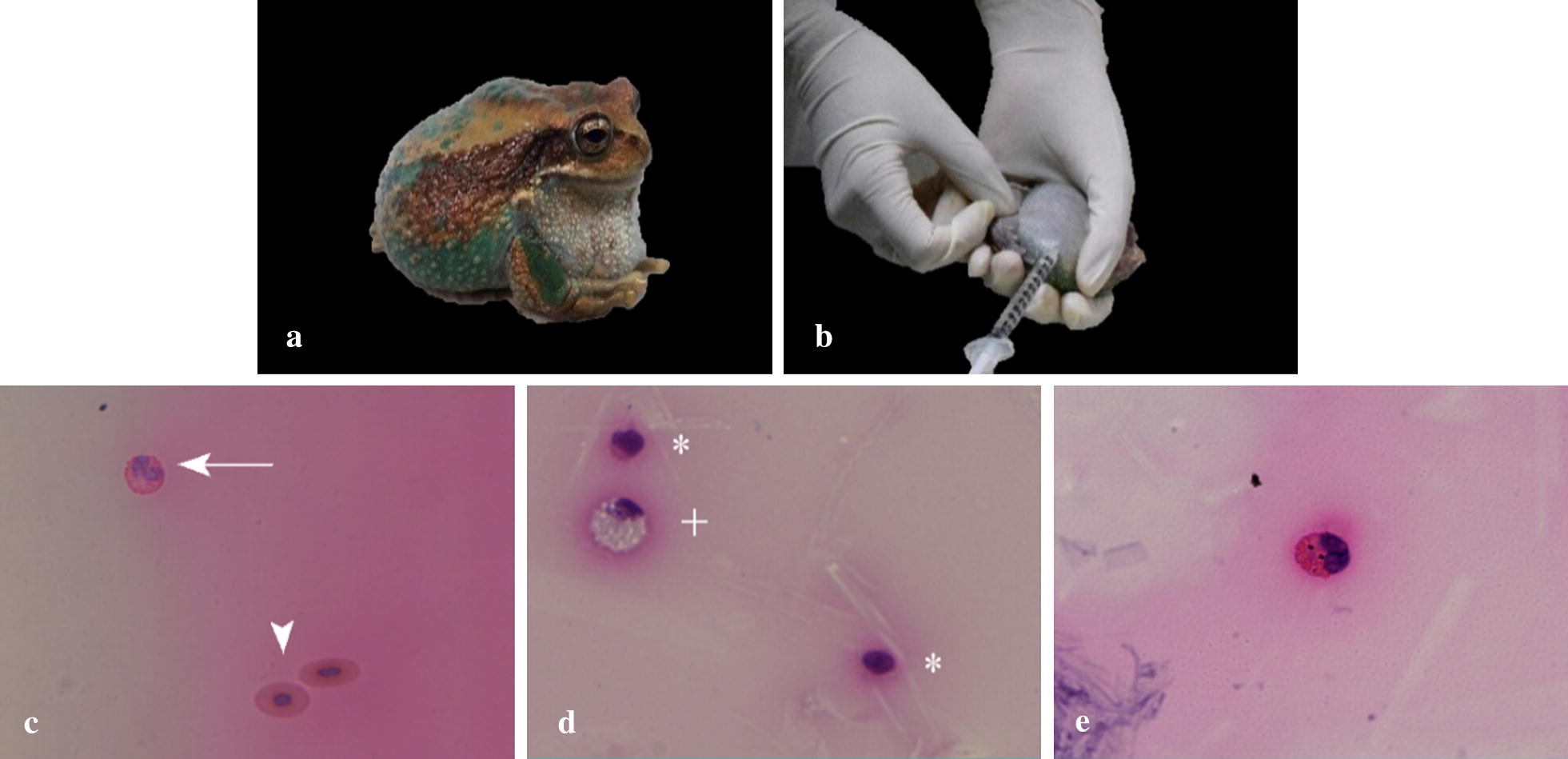
Edema, sampling and morphology of edema fluid cells. *Gastrotheca riobambae* with edema in the coelomic cavity, legs, and arms (**a**). Position of animal in dorsal recumbency for sampling of edema fluid (**b**). Photomicrographs of the cells observed: erythrocytes (arrowhead) and eosinophils (arrow) (**c**), reactive neutrophils (plus sign) and lymphocytes (asterisks) (**d**) and neutrophils with endocytic vesicles visible inside the cytoplasm (**e**). Slides were stained with H&E, ×400 for **c**–**e**

During the time of the study (3 months), the number of individuals was limited by the presence or absence of the syndrome, only anurans of *Gastrotheca* genus had edema syndrome in the institution. Samples were taken from live animals with the syndrome to prevent contamination by autolysis process. Ten males and ten females, with representatives of *G. litonedis* (ten), *G. pseustes* (eight) and *G. riobambae* (two). All animals had obvious edematous swelling in the coelomic cavity, with expansion through their subcutaneous tissue into the arms and legs (Fig. 1a).

All animals lived in captivity, but 85% of the frogs were collected from natural habitats in Ecuador in the provinces of Imbabura, Cotopaxi, Tungurahua, Chimborazo, Cañar, and Azuay between 2007 and 2010. One individual was provided by the Amaru Zoo in Cuenca in 2009, and the remaining 15% were born in the center in 2007, 2009 and 2010. The animals lived in terrariums that contained a substrate made by dried leaves and dirt that were collected from the jungles located in the Ecuadorian Amazon and autoclaved monthly. The humidity was approximately 60–80%, and the temperature inside the terrarium varied from 20 to 22 °C. The animals were given only water filtered with 20-in. thread, washable polyester, and activated charcoal and exposed to UVB (ultraviolet B) light.

#### Sampling

The sampling, handling and preservation of edema fluid from the coelomic cavity was based on descriptions provided in specific literature [18, 19] (Fig. 1b). The animal was placed in dorsal recumbency with a level decline in the cranial direction. The para-medial region was cleaned with diluted chlorhexidine. In that region, an insulin needle was inserted, 1 ml of the liquid was extracted, the needle was carefully removed, and a small pressure was generated in the area with sterile gauze. The sample was divided into two aliquots (0.4 ml each) for microbiological and cytological characterization. All samples were transported in refrigerated conditions and immediately processed in the Laboratory of Microbiology of the Universidad de Las Américas.

#### Microbiological analysis

The materials and methods needed for the analysis of samples were based on similar studies or studies of other species [11, 20–22]. The portion for microbiological characterization was inoculated into tubes containing thioglycolate and resazurin for transport to the laboratory. Incubation was performed at 37 °C in an aerobic environment. Bacteria were isolated on Bi Plate Blood MacConkey agar, and the following laboratory tests were performed: Gram stain (Merck^®^, Germany), catalase (Mediquin^®^, Ecuador), oxidase (Hardy Diagnostics^®^, USA), bile-esculin (HiMedia Laboratories^®^, India) and Microgen ID (Microgen Bioproducts Ltd.^®^, United Kingdom).

#### Cytological analysis

Two smears of the edema fluid per animal were stained using the hematoxylin–eosin staining technique [23]. Subsequently, cell counting and analysis of the characteristics of the cells were performed [11, 24].

#### Statistical analysis

The program Minitab^®^ 17.1.0 was used to obtain the mean, standard deviation and differences in cell type. The Mann–Whitney test was used to determine any significant differences between males and females.

### Results

#### Microbiology

Fourteen bacteria were obtained from 12 amphibians (Table 1); 78% were gram-negative bacilli (*Pasteurella haemolytica*, *Hafnia* (*H*.) *alvei*, *Enterobacter agglomerans*, *Aeromonas* (*A*.) *hydrophila*, *Pseudomonas* (*P*.) *fluorescens*, *Burkholderia pseudomallei*, *Salmonella arizonae*, *Klebsiella ozaenae*, *Klebsiella oxytoca*, *Enterobacter gergoviae*, *Enterobacter sakazakii*, and *Yersinia enterocolitica*), and 22% were gram-positive cocci (*Streptococcus* spp. and, *Enterococcus* spp.). The bacterial growth of the samples from females was mostly pure cultures, while most of the samples from males exhibited mixed growth.

**Table 1:** Gram stain characteristics and macroscopic aspects of bacteria isolated from *Gastrotheca* species

Sex/species	Isolates	Gram stain Positive or negative	Gram stain Morphotypes
Female *G. litonedis* (n = 4/10)	*Pasteurella haemolytica*	−	Bacilli
	*Hafnia alvei*	−	Bacilli
	*Enterobacter agglomerans*	−	Bacilli
	*Aeromonas hydrophila*/*Pseudomonas fluorescens*	−/−	Bacilli/Bacilli
Male *G. litonedis* (n = 2/10)	*Streptococcus*	+	Cocci
	*Burkholderia pseudomallei*/*Salmonella arizonae*	−/−	Bacilli/Bacilli
Female *G. pseustes* (n = 2/8)	*Klebsiella ozaenae*	−	Bacilli
	*Enterobacter gergoviae*/*Enterococcus* spp.	−/+	Bacilli/Cocci
Male *G. pseustes* (n = 2/8)	*Enterobacter sakazakii*/*Enterococcus* spp.	−/+	Bacilli/Cocci
	*Yersinia enterocolitica*	−	Bacilli
Male *G. riobambae* (n = 2/2)	*Klebsiella oxytoca*/*Enterococcus* spp.	−/+	Bacilli/Cocci
	*Klebsiella ozaenae*/*Burkholderia pseudomallei*	−/−	Bacilli/Bacilli

#### Cytology

Approximately 30% of the total analyzed samples did not show cellular components, while the remaining 70% did. Normal morphology of some identified cells could be seen, and some reactive neutrophils were observed (Fig. 1c–d). Similarly, phagocyte vacuoles were observed in the leukocytes of one individual (Fig. 1e). No other type of cells, such as mesothelial cells, were found.

The cells observed in females and males did not exhibit significant differences between the relationship of the cell count and the sex of the individual. Females had higher values of erythrocytes, neutrophils, basophils, and monocytes than males. On the other hand, males had higher values of eosinophils, lymphocytes, and thrombocytes (Table 2).

**Table 2:** Erythrocyte and leukocyte series of edema fluid from 20 individuals of the genus *Gastrotheca*

Cell type (%)	Females (n = 10)	Males (n = 10)
Erythrocytes	23.5 ± 27.7 (0.0–73.0)	17.5 ± 24.1 (0.0–61.0)
Neutrophils	4.2 ± 4.7 (0.0–13.5)	2.8 ± 3.9 (0.0–12.5)
Eosinophils	0.5 ± 0.7 (0.0–2.0)	1.6 ± 2.3 (0.0–7.0)
Basophiles	2.5 ± 5.4 (0.0–17.5)	0.4 ± 0.8 (0.0–2.5)
Lymphocytes	37.7 ± 31.4 (0.0–80.5)	46.4 ± 36.9 (0.0–91.0)
Monocytes	0.3 ± 0.4 (0.0–1.0)	0.1 ± 0.2 (0.0–0.5)
Thrombocytes	1.4 ± 1.5 (0.0–4.0)	1.5 ± 1.1 (0.0–3.0)

Values are expressed as the mean ± standard deviation (minimum–maximum)

### Discussion

#### Microbiology

When evaluating a bacterial infection, it must be considered that the presence of bacteria in a culture does not always indicate disease since these can be bacteria of the individual’s normal microbiota [25, 26].

Most bacterial septicemias are caused by aerobic gram-negative bacteria, although there are some reports of septicemia caused by gram-positive bacteria [13]. This statement coincides with the findings of the present study, in which 78% of the bacteria identified were gram negative and the remaining 22% were gram positive.

The potentially pathogenic bacteria identified agree with those detailed in the literature on amphibians: *Streptococcus* [27], *Klebsiella* spp. [14, 27], *H. alvei* [27, 28], *Pseudomonas* spp. [10, 27], *Enterobacter* spp. [22], *A. hydrophila* [15, 29], *Salmonella* spp. [30], and *Enterococcus* spp. [31]. However, not all bacteria detected have pathogenic potential. A previous study detected cultivable bacteria in *G. riobambae* and *G. plumbea* associated with the skin of these frogs in Ecuador and found several isolates with known anti-Bd activity (*P. fluorescens* and *A. hydrophila*) and other genera and species similar to those reported in our study (e.g., *Enterobacter* spp., *Burkholderia cepacia*, *Pseudomonas palleroniana* and *Aeromonas veronii*) [32].

Although most of the bacteria in the present study have been described as typical of the amphibian microbiota, they could be part of a secondary infection caused by a primary viral or fungal infection [11, 22, 26, 27]. *A. hydrophila*, *Pseudomonas* spp. and *Streptococcus* spp. have been identified as the cause of red-leg syndrome [33].

#### Cytology

In the case of the genus *Gastrotheca*, cytology reference values do not exist. Additionally, the morphology of the cells varies between anuran species and is usually compared to that of other vertebrates [11, 24, 34, 35].

It is necessary to mention that in amphibians, knowledge of the response of the leukocyte series to diseases is limited, partly because leukocyte evaluation is not a routine methodology used in anurans [34] and because the cells identified in the edema fluid samples came from a blood extravasation, not from peripheral blood [36].

Studies show that the red blood cell count in females is lower than that in males and that the leukocyte count is uniform [37]. In general, normal cytology values and values with variations derived from pathologies can be influenced by sex [38]. In this case, females had a higher percentage of erythrocytes, neutrophils, basophils, and monocytes, and males had a higher percentage of lymphocytes and eosinophils; however, no relationship was found between the statistical results analyzed and the sex of the individuals.

The basophil count was low; these cells play a similar function to those mammals because they are peroxidase positive and have hydrolytic enzymes [34]. Similarly, thrombocytes were found in small quantities, and these cells have a function comparable to the function of those of reptiles and birds [24, 39]. Eosinophils also exhibited low counts; these cells are active in parasitic infections or in polluted areas [40, 41], but they have an inferior phagocytosis ability [34]. These low numbers are observed even in noninflammatory effusions, where there are low counts of mesothelial cells, macrophages, granulocytes, and lymphocytes [11].

Neutrophils and monocytes of amphibians share similar characteristics of migration and phagocytic activity and have the same enzymes that occur in the same cells of other vertebrates, and an increased count suggests an inflammatory response [34]. Only neutrophils are present in high numbers in both sexes of *Gastrotheca*.

B lymphocytes are capable of phagocytosis in response to bacterial infections [42]. In the present study, it was possible that there was a relationship between the results obtained in the microbiological characterization and the lymphocytosis and neutrophilia observed due to the presence of bacteria.

Likewise, a study carried out on individuals from the herpetofauna of Turkey determined that 80% of the leukocytes present in peripheral blood were lymphocytes and monocytes [43]. These results are partially compatible with the results regarding lymphocytes of our study. Their presence is related to an excitement response, stimulation of the immune system, or possibly lymphoid leukemia [34].

Despite the presence of the bacteria and types of cells in this study, all the animals did not show evidence or clinical signals congruent with infectious disease. In this case, it may be a consequence of captive management; since amphibians are demanding in terms of environmental and nutritional requirements, those factors most likely increased the number of bacteria but did not cause disease or death. Systemic bacterial infections are common in debilitated, stressed, or crowded amphibians [11].

One kind of effusion fluid type is septic exudate within phagocytes [21]. In amphibians and reptiles, the inflammatory response depends on the temperature and is classified according to the predominant cell type [39]. In addition, if phagosomes or vacuoles from phagocytosis are observed in leukocytes due to phagocytosis of bacteria, septic inflammation is considered [40]. In contrast, samples with higher total protein (> 3 g/dl) and cell counts (> 7000/ml) are likely exudates, and inflammatory conditions should be considered [11]. A unique sample was observed with one phagosome, but it is necessary to add information such as specific gravity, total protein, and cell count to categorize the type of cavity fluid.

Another type of fluid is the transudate type that most likely derives from metabolic imbalance, such as hypoproteinemia [44]. The increase in solute attracts solvent, in this case, water, which leads to a weight increase. Here, it is possible to consider edema as a chronic process, which facilitates the passage of water over a large time period and, consequently, an increase in weight and abdominal fluid with low densities.

It is important to understand that edema syndrome has a strong relation to a deficit in osmoregulation, which could be caused by many pathogens and failure of a variety of body systems, causing difficulty in antemortem diagnosis [9]. In this study, the cause of edema was most likely related to a fusion of chronic osmoregulation failure and pathogens. Other causes of edema are a low solute concentration in water or acute renal disease (captivity) [45]. Thus, additional studies on the level of management and pathology are necessary to complement a microbiological and cytological characterization of this problem in captive anurans.

## Limitations

The literature on the normal microbiota and cytological characteristics of marsupial frogs is limited. The design of this study is adequate only for a preliminary investigation since complementary approaches will be necessary as: characterization of other potential microorganism as virus or fungi, physicochemical characterization of fluid, if possible samples of fluid coelomic and comparison of blood parameters of non-diseased animals, and sequencing of the isolated bacteria.

## Data Availability

All relevant data are included in the manuscript.

## Abbreviations

*G*: *Gastrotheca*
UVB: ultraviolet B
*H*: *Hafnia*
*A*: *Aeromonas*
*P*: *Pseudomonas*
